# Comparative analysis data of SF1 and SF2 helicases from three domains of life

**DOI:** 10.1016/j.dib.2017.02.047

**Published:** 2017-03-03

**Authors:** Wafi Chaar, Hiba Ibrahim, Juliana Kozah, Hala Chamieh

**Affiliations:** a Hloul Business Analytics, Omar Daouk Street, Beirut, Lebanon; b Beirut Arab University, Faculty of Science, Tripoli, Lebanon; c Université Saint Esprit, Jounieh, Lebanon; d Azm Center for Research in Biotechnology and its Applications, Lebanese University, Lebanon; e Department of Biology, Lebanese University, Faculty of Science, Tripoli, Lebanon

**Keywords:** Helicase, Archaea, SF1, SF2, Phylogenetics

## Abstract

SF1 and SF2 helicases are important molecular motors that use the energy of ATP to unwind nucleic acids or nucleic-acid protein complexes. They are ubiquitous enzymes and found in almost all organisms sequenced to date. This article provides a comparative analysis for SF1 and SF2 helicase families from three domains of life archaea, human, bacteria. Seven families are conserved in these three representatives and includes Upf1-like, UvrD-like, Rad3-like, DEAD-box, RecQ-like. Snf2 and Ski2-like. The data highlight conservation of the helicase core motifs for each of these families. Phylogenetic analysis presented on certain protein families are essential for further studies tracing the evolutionary history of helicase families. The data supplied in this article support publication “Genome-wide identification of SF1 and SF2 helicases from archaea” (Chamieh et al., 2016) [1].

**Specifications Table**

**Table:** 

Subject area	*Biology*
More specific subject area	*Genomics, Phylogenetics, helicase, archaea*
Type of data	*Figures*
How data was acquired	*Computational analysis*
Data format	*Analyzed*
Experimental factors	*Protein sequences were retrieved from online databases and used for detection of protein domain conservation and Phylogenetic analysis.*
Experimental features	*Human, E.coli and archaea protein helicase sequences were aligned using TCOFFEE or PROMALS3D..Conserved motifs were detected from multiple sequence alignments using WebLOGO software. Phylogenetic analysis were performed using Maximum Likelihood Methods or Bayesian Methods after protein alignment trimming by TrimAl.*
Data source location	*Lebanese University*
Data accessibility	Data is available within this article

**Value of the data**

• The presented data on highly conserved amino acids in each of the seven conserved families across the three domains of life is important to design mutagenic studies and therefore determine functional conservation required for helicase function.
• Protein sequence comparison between SF1 and SF2 helicase families will allow establishing key experiments for genetic and biochemical analysis of helicase action.
• Phylogenetic tree data of Upf1-like, ski2-like and rad3-like shed light on the phylogenic relationship between these helicases in archaea, human and *E.coli*. The data offers valuable information on the complex evolutionary history within a helicase family and is a starting point for more detailed evolutionary studies on helicase subfamilies.

## 1 Data

**Fig. 1:**
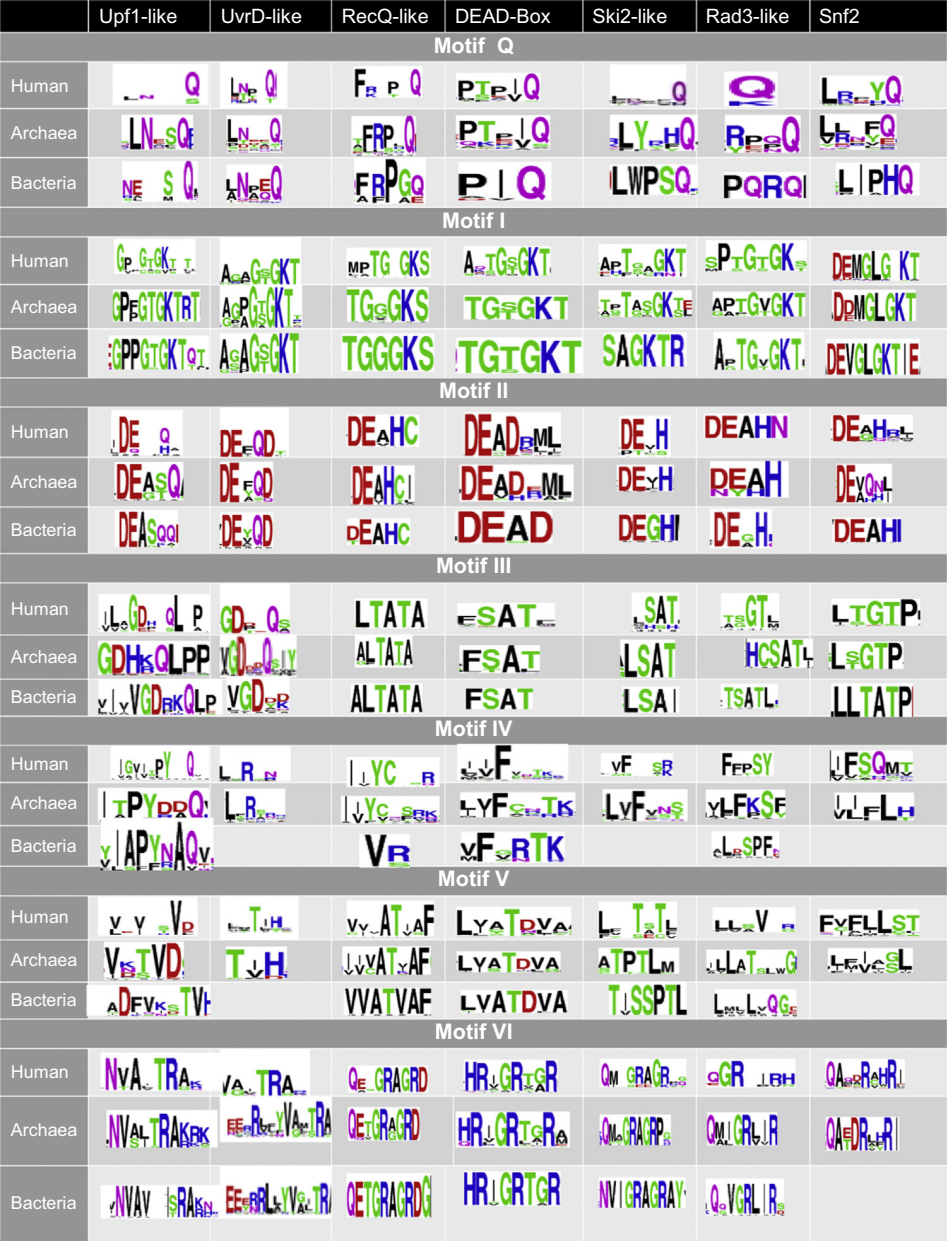
Conserved motifs of the helicase core domain for SF1 and SF2 families across the three domains. All protein sequences were retrieved from existing protein databases. Multiple protein sequence alignment was performed using T-COFFEE EXPRESSO program for small sequence numbers (<150 sequences) (2) or PromalS3D for large sequence numbers (>150 sequences). Conserved motifs were generated from the multiple sequence alignment files for protein sequences within the same family using the WEBLOGO software.

**Fig. 2:**
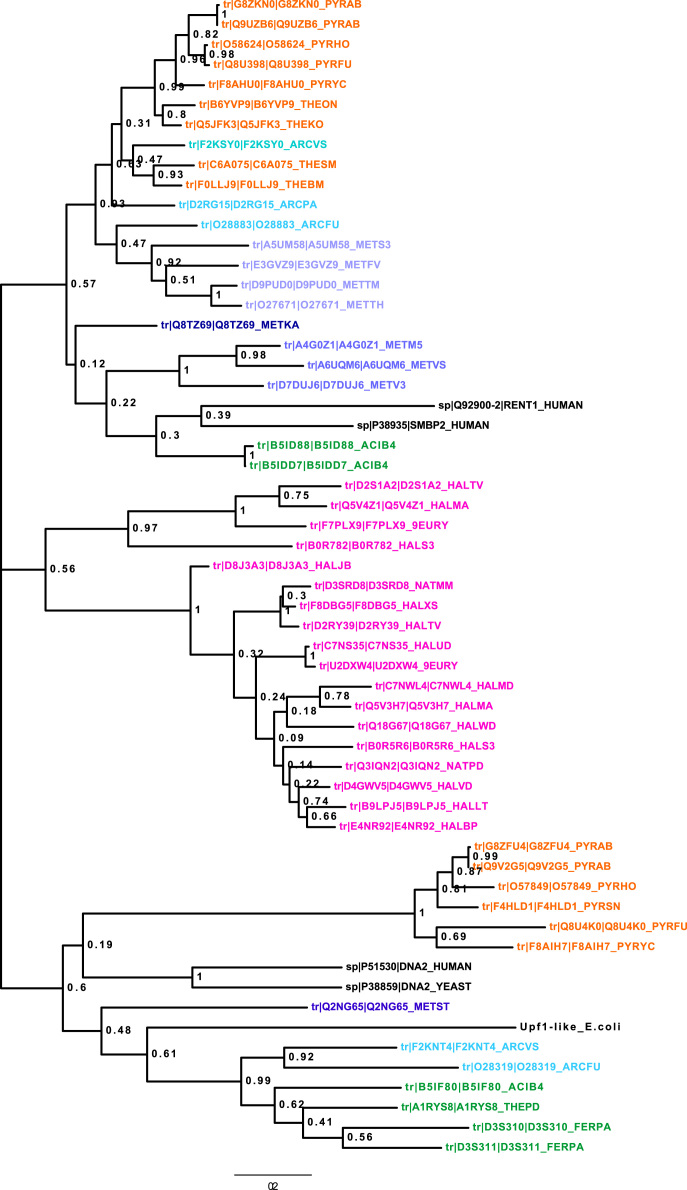
Molecular Phylogenetic analysis of the Upf1-like family by Maximum Likelihood method. The evolutionary history was inferred by using the Maximum Likelihood method based on the Whelan And Goldman+Freq. model (WAG+F). The percentage of trees in which the associated taxa clustered together is shown next to the branches. The analysis involved 58 amino acid sequences. All positions containing gaps and missing data were eliminated. There were a total of 230 positions in the final dataset. Evolutionary analyses were conducted in MEGA7.

**Fig. 3:**
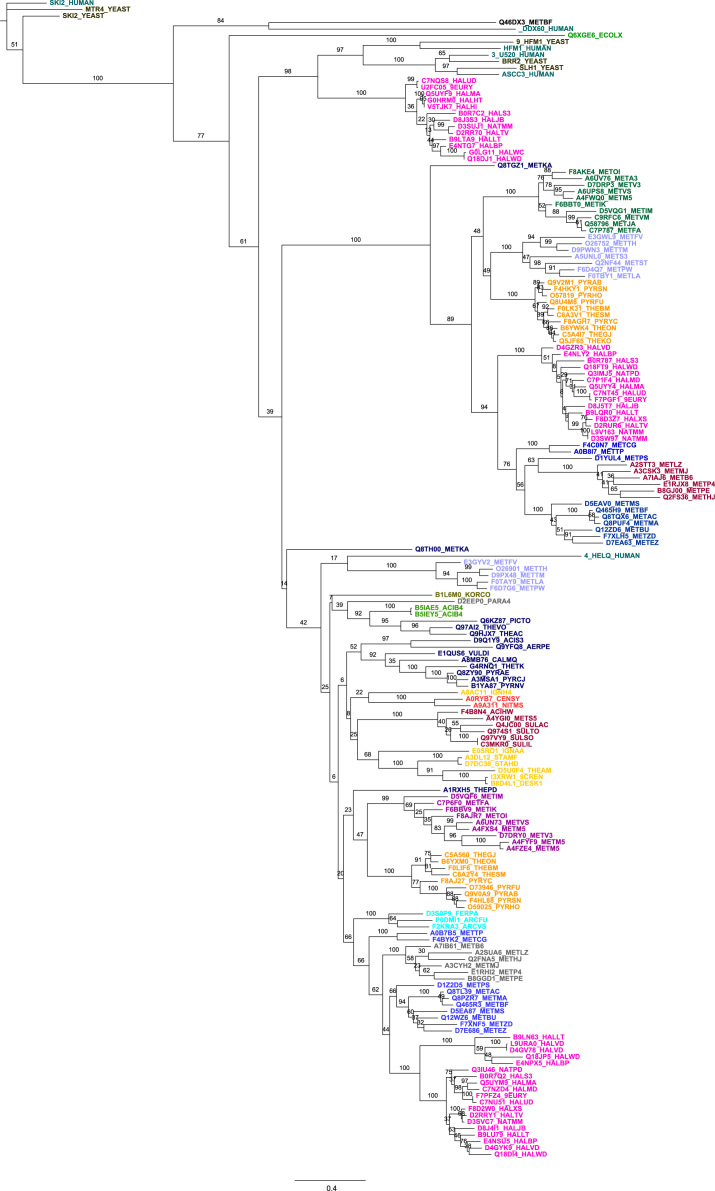
Molecular Phylogenetic analysis of Ski2-like family by Bayesian Method. The evolutionary history was inferred by using the Bayesian method based on the MTMam model. The analysis involved 178 amino acid sequences. Evolutionary analyses were conducted in MrBayes. Two runs of 750,000 generations were conducted. Burn-in was set to 25%. Robustness of nodes was assessed with Bayesian posterior probabilities.

**Fig. 4:**
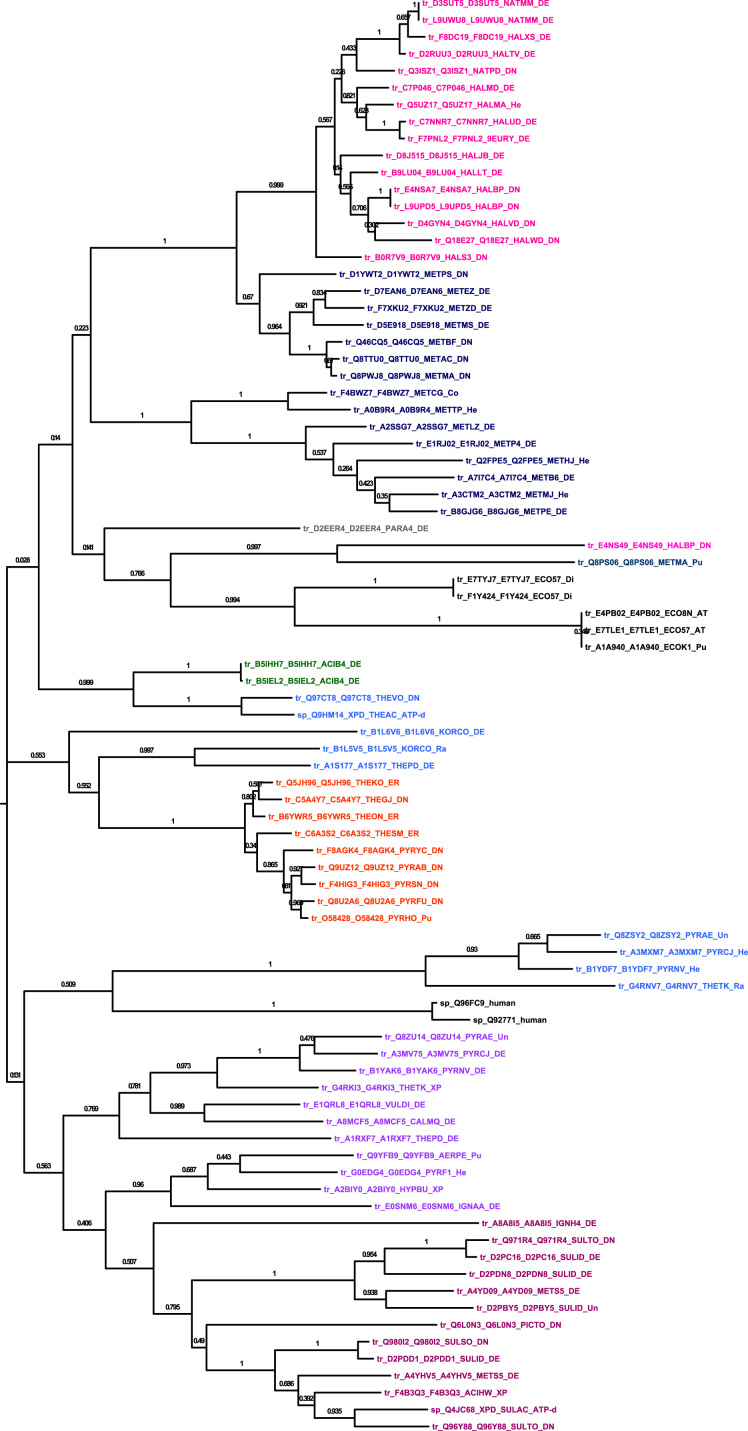
Molecular Phylogenetic analysis of rad3-like family by Maximum Likelihood method. The evolutionary history was inferred by using the Maximum Likelihood method based on the WAG+F model. The percentage of trees in which the associated taxa clustered together is shown next to the branches. The analysis involved 85 amino acid sequences. All positions containing gaps and missing data were eliminated. There were a total of 268 positions in the final dataset. Evolutionary analyses were conducted in MEGA7.

Four figure files are presented. Fig. 1 denotes a comparative analysis of helicase core motifs in conserved families from archaea, bacteria and human. Fig. 2, Fig. 3, Fig. 4 are phylogenetic trees obtained after Maximum Likelihood analysis for Upf1-like and Rad3-like families, and Bayesian analysis for ski2-like helicase family.

## 2 Experimental design, materials and methods

All protein sequences were retrieved from existing protein databases and were used with their UniProt accession numbers and were classified into different families as shown in Chamieh et al. [1], [2]. Multiple protein sequence alignment was performed using T-COFFEE EXPRESSO program for small sequence numbers (<150 sequences) [3] or PromalS3D for large sequence numbers (>150 sequences) [4]. Fig. 1 was obtained from the multiple sequence alignment files for protein sequences within the same family using the WEBLOGO software [5]. Sequences were inspected for their correct alignment within the helicase core domain. Multiple sequence alignment was trimmed using TrimAl v1.3 method set to automated [6]. The best evolutionary fit model was identified using ProtTest [7]. Phylogenetic analysis was performed using Maximum Likelihood analysis from MEGA7 software [8] or MrBayes with the TOPALI platform [9], [10].
