# Supplementary material for: Comparative analysis data of SF1 and SF2 helicases from three domains of life
Source: Data Brief. 2017 Mar 3;11:510–6. doi: 10.1016/j.dib.2017.02.047 (PMC5349461; doi:10.1016/j.dib.2017.02.047)
Supplement: Supplementary file 1 — Supplementary material [file mmc1.docx]

**Conflict of interest**

The authors whose names are listed immediately below certify that they have NO affiliations with or involvement in any organization or entity with any financial interest (such as honoraria; educational grants; participation in speakers’ bureaus; membership, employment, consultancies, stock ownership, or other equity interest; and expert testimony or patent-licensing arrangements), or non-financial interest (such as personal or professional relationships, affiliations, knowledge or beliefs) in the subject matter or materials discussed in this manuscript.
